# Supplementary material for: Ionized calcium level at emergency department arrival is associated with return of spontaneous circulation in out-of-hospital cardiac arrest
Source: PLoS One. 2020 Oct 12;15(10):e0240420. doi: 10.1371/journal.pone.0240420 (PMC7549779; doi:10.1371/journal.pone.0240420)
Supplement: S1 Table — (DOCX) [file pone.0240420.s001.docx]

**S1 Table. Correlation between ionized calcium concentration and survival discharge and favourable neurologic outcome by multivariable logistic regression test**

| Outcomes | Odds ratio | 95% CI | *P* value |
| --- | --- | --- | --- |
| Survival discharge^†‡^ | 0.99 | 0.72-1.36 | 0.948 |
| Favourable neurologic outcome^†‡^ | 0.45 | 0.03-6.55 | 0.560 |

CI, confidence interval.

^†^Each value was obtained by multivariate logistic regression test based on statistically significance in univariable logistic regression test.

^‡^Adjusted for age, sex, witness of cardiac arrest, bystander CPR, total CPR duration, estimated time from collapse to ED arrival, total administered epinephrine dose, albumin, arterial pH, phosphorus, blood urea nitrogen, creatinine, potassium, and magnesium levels.
