# Supplementary material for: Ionized calcium level at emergency department arrival is associated with return of spontaneous circulation in out-of-hospital cardiac arrest
Source: PLoS One. 2020 Oct 12;15(10):e0240420. doi: 10.1371/journal.pone.0240420 (PMC7549779; doi:10.1371/journal.pone.0240420)
Supplement: S2 Table — (DOCX) [file pone.0240420.s002.docx]

**Supplementary_table 2. The correlation analysis between total administered dose of epinephrine and the ionized calcium**

| Category | | Total administered dose of epinephrine | Ionized calcium |
| --- | --- | --- | --- |
| Total administered dose of epinephrine | Pearson’s correlation coefficient  p-value | 1.00 | -0.0135  0.700 |
| Ionized calcium | Pearson’s correlation coefficient  p-value | -0.0135  0.700 | 1.00 |
