# Supplementary figures and images for: Ionized calcium level at emergency department arrival is associated with return of spontaneous circulation in out-of-hospital cardiac arrest
Source: PLoS One. 2020 Oct 12;15(10):e0240420. doi: 10.1371/journal.pone.0240420 (PMC7549779; doi:10.1371/journal.pone.0240420)

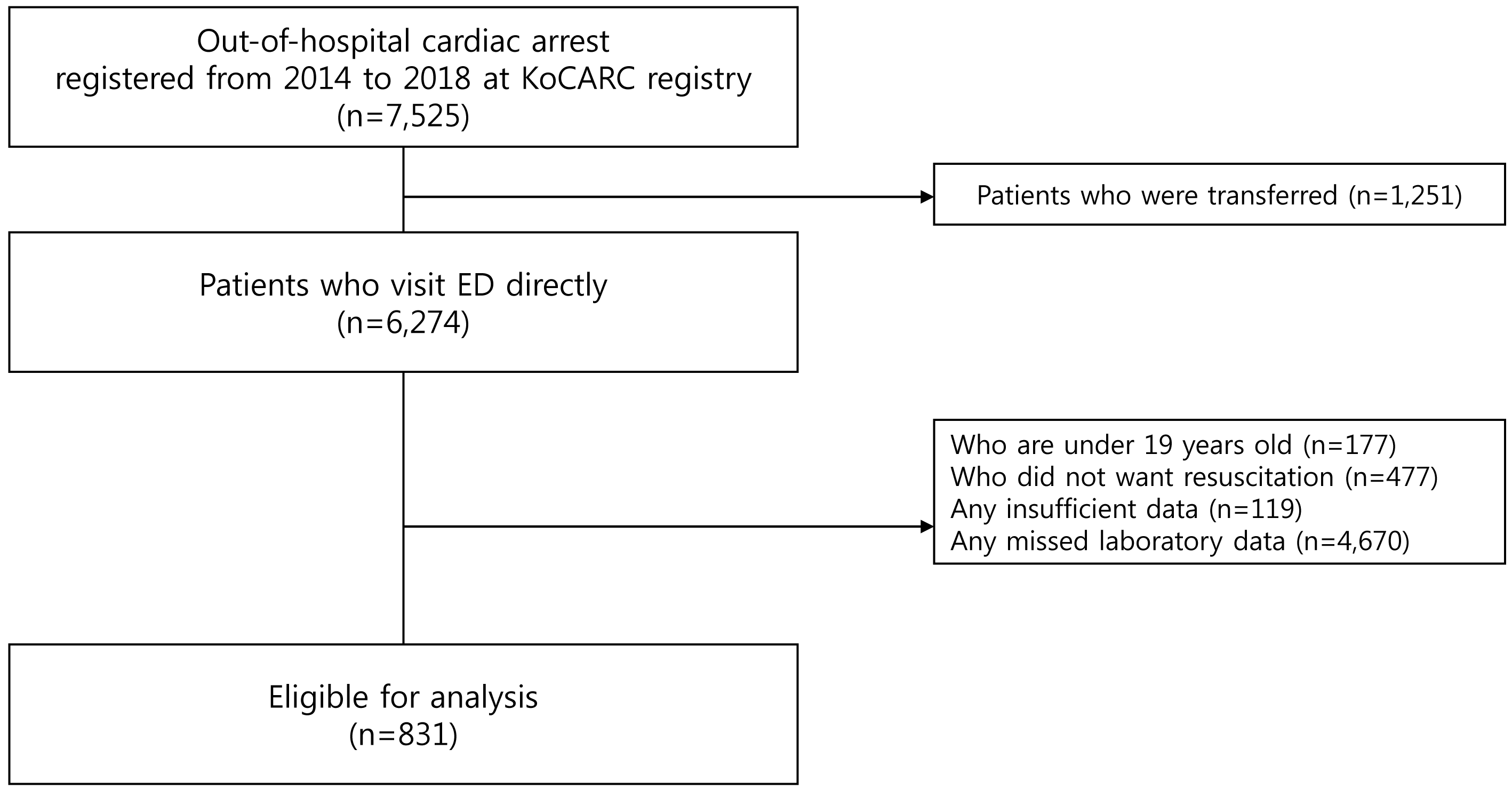

Supplement: S1 Fig — *KoCARC: Korean Cardiac Arrest Research Consortium data. (TIF) [file pone.0240420.s003.tif]

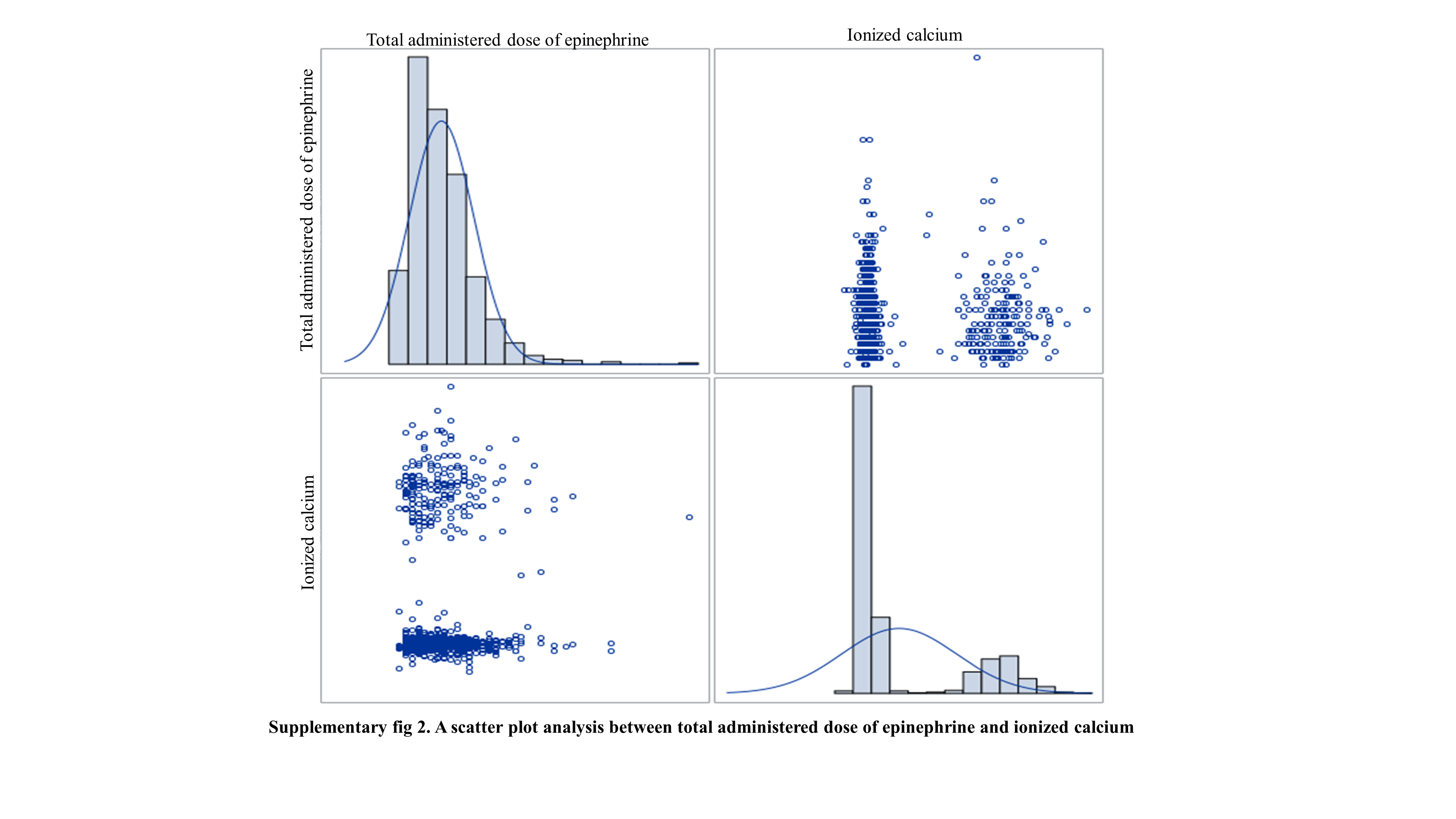

Supplement: S2 Fig — (TIF) [file pone.0240420.s004.tif]
