# Supplementary material for: Ionized calcium level at emergency department arrival is associated with return of spontaneous circulation in out-of-hospital cardiac arrest
Source: PLoS One. 2020 Oct 12;15(10):e0240420. doi: 10.1371/journal.pone.0240420 (PMC7549779; doi:10.1371/journal.pone.0240420)
Supplement: Attachment — Submitted filename: Response to Reviewers.docx [file pone.0240420.s005.docx]

**Response to reviewers**

We appreciate your kind recommendations for improving the quality of our manuscript. Here we present our responses or revised content corresponding to your suggestions.

**Comment 1. Please ensure that your manuscript meets PLOS ONE's style requirements, including those for file naming.**

Answer 1: We checked it once more and confirmed the style requirement.

**Comment 2. In ethics statement in the manuscript and in the online submission form, please provide additional information about the patient records used in your retrospective study. Specifically, please ensure that you have discussed whether all data were fully anonymized before you accessed them and/or whether the IRB or ethics committee waived the requirement for informed consent. If patients provided informed written consent to have data from their medical records used in research, please include this information.**

Answer 2: We added the comments about data coding and informed consent as follows;

*Patient information was coded as anonymous so that researchers could not recognize the patient’s personal information.*

*This study protocol was approved by the Institutional Review Board of Wonju Severance Christian Hospital (IRB No.CR319065) and informed consent was waived in case of unsuccessful resuscitation and obtained after intensive care unit admission in case of successful resuscitation.*

**Comment 3. Thank you for stating the following in the Acknowledgments Section of your manuscript:**

**"This consortium was supported by the Korea Centers for Disease Control and Prevention
during the organizing stage. Currently, the KoCARC is partly supported by the Korean
Association of Cardiopulmonary Resuscitation."**

**We note that you have provided funding information that is not currently declared in your Funding Statement. However, funding information should not appear in the Acknowledgments section or other areas of your manuscript. We will only publish funding information present in the Funding Statement section of the online submission form.**

**Please remove any funding-related text from the manuscript and let us know how you would like to update your Funding Statement. Currently, your Funding Statement reads as follows:**

**"The authors received no specific funding for this work."**

Answer 3: We removed the funding statement from Acknowledgement section and added it in online submission form. We revised the comments about Korea Centers for Disease Control and Prevention because the institution supported administrative work, not a fund. The revised comment is as follows;

The Korean Cardiac Arrest Research Consortium was supported administratively by the Korea Centers for Disease Control and Prevention during the organizing stage.

**Comment 4. We note that you have indicated that data from this study are available upon request. PLOS only allows data to be available upon request if there are legal or ethical restrictions on sharing data publicly. For information on unacceptable data access restrictions, please see** [**http://journals.plos.org/plosone/s/data-availability#loc-unacceptable-data-access-restrictions**](http://journals.plos.org/plosone/s/data-availability#loc-unacceptable-data-access-restrictions)**.**

**In your revised cover letter, please address the following prompts:**

**a) If there are ethical or legal restrictions on sharing a de-identified data set, please explain them in detail (e.g., data contain potentially identifying or sensitive patient information) and who has imposed them (e.g., an ethics committee). Please also provide contact information for a data access committee, ethics committee, or other institutional body to which data requests may be sent.**

**b) If there are no restrictions, please upload the minimal anonymized data set necessary to replicate your study findings as either Supporting Information files or to a stable, public repository and provide us with the relevant URLs, DOIs, or accession numbers. Please see** [**http://www.bmj.com/content/340/bmj.c181.long**](http://www.bmj.com/content/340/bmj.c181.long) **for guidelines on how to de-identify and prepare clinical data for publication. For a list of acceptable repositories, please see** [**http://journals.plos.org/plosone/s/data-availability#loc-recommended-repositories**](http://journals.plos.org/plosone/s/data-availability#loc-recommended-repositories)**.**

**We will update your Data Availability statement on your behalf to reflect the information you provide.**

Answer 4: We added the following policy for data access in the cover letter.

**Comment 5. One of the noted authors is a group or consortium [Korean Cardiac Arrest Research Consortium (KoCARC) Investigators]. In addition to naming the author group and listing the individual authors and affiliations within this group in the acknowledgments section of your manuscript, please also indicate clearly a lead author for this group along with a contact email address.**

Answer 5: We have indicated a chairman for our KoCARC registry with a contact e-mail address as follows;

We would like to acknowledge the chairman of the KoCARC: Sung Oh Hwang (Yonsei University Wonju College of Medicine, e-mail address: [shwang@yonsei.ac.kr](mailto:shwang@yonsei.ac.kr))

**Comment 6. Please include a caption for figure 1.**

Answer 6. We added a caption for fig 1.

Comment 7. Review Comments to the Author

Please use the space provided to explain your answers to the questions above. You may also include additional comments for the author, including concerns about dual publication, research ethics, or publication ethics. (Please upload your review as an attachment if it exceeds 20,000 characters)

Reviewer #1: It's mine opinion that it's an interesting study that deserves further investigation useful to define the causes of no difference in terms of mortality.
Evaluate treatment with calcium administration during cardiac arrest.

Reviewer #2: The authors have presented and addressed a potential important point in the field of cardiopulmonary resuscitation where many uncertainty are still to be determined.
The paper is well written and message is clear with some practical insights.


Minor comments

**7-1: Introduction line 63
might be misunderstood
Please change to “misled” or "misinterpreted" or "should be contextualised"**

Answer 7-1: Thank you for your comment. We corrected word as you recommended.

**Comment 7-2: Line 70
Change “would” with “could”**

Answer 7-2: Thank you for your comment. We corrected word as you recommended.

**Comment 7-3: “Relationship between survival to discharge and favorable neurologic outcome and ionized calcium level”
It is hard to think that the first measurement of ionized calcium level in patients with ROSC may affect the outcome of the post-cardiac arrest syndrome. I would add this concept in the discussion.**

Answer 7-3) Thank you very much for commenting on what we were worried about. As you mentioned, it is difficult to evaluate the predictability of the ionized calcium drawn at ED arrival for survival discharge or favorable neurologic outcome because there was high risk of bias from patient’s physiologic or pathologic status and treatment modalities or responsibility during post-cardiac arrest care. We removed the description about the relationship between ionized calcium and survival discharge or favorable neurologic outcome at first paragraph on discussion section and the related description was left on discussion section, line 233 through 243.

**Comment 7-4: Is it known How exogenous adrenaline administration affect ionized calcium? If yes, and the data are reliable, this should be referenced otherwise it could be a point for further analysis/study.**

Answer 7-4: Thank you for your important comments. For confirming your suggestion, we drew a scatter plot and performed a correlation analysis between total administered dose of epinephrine and ionized calcium.

In a scatter plot, there is no linear correlation between the two variables (Supplementary _ fig 2).

As a result of the correlation analysis, it was analyzed that there was a negative correlation between the two variables, but the correlation coefficient was close to 0, so there was little correlation between the two variables (R=-0.0135) (Supplementary_table 2).

There were some studies [1-3] on the relationship between administration of epinephrine and ionized calcium in non-cardiac arrest situation, but we cannot find a study in cardiac arrest situation.

Judging from descriptions above, it seems hard to believe that there is any correlation between total administered dose of epinephrine and the ionized calcium.

We added the above results on the supplements.

Reference

1.	Kenny, A.D., *Effect of catecholamines on serum calcium and phosphorus levels in intact and parathyroidectomized rats.* Naunyn-Schmiedebergs Archiv für experimentelle Pathologie und Pharmakologie, 1964. **248**(2): p. 144-152.

2.	Ljunhgall, S., et al., *Effects of epinephrine and norepinephrine on serum parathyroid hormone and calcium in normal subjects.* Exp Clin Endocrinol, 1984. **84**(3): p. 313-8.

3.	Musso, E. and M. Vassalle, *Effects of norepinephine, calcium, and rate of discharge on 42K movements in canine cardiac Purkinje fibers.* Circulation research, 1978. **42**(2): p. 276-284.
